# An Endless Riddle of Wide Complex Tachycardia: Ventricular Tachycardia (VT) or Supraventricular Tachycardia (SVT)?

**DOI:** 10.7759/cureus.109414

**Published:** 2026-05-22

**Authors:** Narut Prasitlumkum, Suphatcha Kittiwongsopon, Pattara Rattanawong

**Affiliations:** 1 Cardiology, Hawaii Pacific Health, Honolulu, USA; 2 Anesthesiology, Siriraj Hospital, Mahidol University, Bangkok, THA

**Keywords:** 12-lead ecg, lead misplacement, radio-frequency ablation, supraventricular tachycardia (svt), ventricular dysrhythmia

## Abstract

Wide complex tachycardia (WCT) presents a diagnostic challenge, particularly in distinguishing ventricular tachycardia (VT) from supraventricular tachycardia (SVT) with aberrancy. We report an 81-year-old man with recurrent WCT initially suspected to be VT based on clinical and electrocardiographic features. However, inconsistencies across serial electrocardiographic tracings limited diagnostic certainty. Specifically, variations in polarity across all limb leads, except aVR and the precordial leads, raised suspicion for limb lead misplacement, particularly left arm-left leg reversal. This electrocardiographic perplexity further complicated the clinical interpretation. To clarify the underlying arrhythmia mechanism, an electrophysiology (EP) study was performed, which demonstrated inducible tachycardia with simultaneous atrioventricular activation (VA = 0), consistent with typical atrioventricular nodal reentrant tachycardia (AVNRT) with aberrancy. Successful slow pathway ablation rendered the arrhythmia noninducible, and the patient has remained free of recurrent arrhythmia during five months of follow-up. This case highlights the limitations of ECG-based algorithms and the importance of careful ECG interpretation and invasive evaluation.

## Introduction

Wide complex tachycardia (WCT) represents a common yet challenging diagnostic scenario in clinical electrophysiology (EP), where distinguishing ventricular tachycardia (VT) from supraventricular tachycardia (SVT) with aberrant conduction is critical for appropriate management. Although several electrocardiographic algorithms have been proposed, their application may be limited in cases with overlapping or atypical features [1-7]. In such situations, reliance on surface electrocardiogram (ECG) interpretation alone may be misleading. A systematic approach integrating clinical context and comparative ECG analysis is essential. On a case-by-case basis, invasive EP assessment may be warranted to establish a definitive diagnosis when diagnostic uncertainty persists despite comprehensive noninvasive evaluation. This case highlights the complexity of WCT interpretation and underscores the importance of integrating multiple diagnostic modalities to arrive at the correct diagnosis.

## Case presentation

An 81-year-old man with a past medical history of prior myocardial infarction status post right coronary artery drug-eluting stent placement a year ago and known prior preserved left ventricular ejection fraction, diabetes mellitus, chronic kidney disease, and hypertension was referred to the EP service for evaluation and management of a WCT, raising concern for VT.

The patient initially presented to the emergency department (ED) with chest tightness and palpitations. His vital signs were hemodynamically stable, except for a maximum heart rate of 150 bpm. The physical examination demonstrated an alert, awake, and fully oriented elderly man, without an abnormal breathing pattern, chest and cardiac auscultation, along with the absence of central and peripheral edema. His jugular venous pressure appears flat without the obvious “frog sign,” which was described in prior literature as a classic sign of atrioventricular nodal reentrant tachycardia (AVNRT) [8].

Laboratory results demonstrated stable baseline kidney function and were otherwise within normal limits across hematologic parameters, basic metabolic panel, liver function tests, and coagulation studies, as shown in Table 1. His echocardiogram demonstrated preserved ejection fraction (55-60%) without abnormal wall motion, and with the absence of valvular abnormalities. Further, he underwent coronary angiography, which showed no significant coronary artery obstruction.

**Table 1:** Laboratory results on presentation, including hematologic parameters, basic metabolic panel, renal function, liver function tests, and coagulation profile. WBC: white blood cell count; PT: prothrombin time; PTT: partial thromboplastin time; BUN: blood urea nitrogen; A/G ratio: albumin/globulin ratio; AST (SGOT): aspartate aminotransferase (serum glutamic-oxaloacetic transaminase); ALT (SGPT): alanine aminotransferase (serum glutamic-pyruvic transaminase); Alk Phos: alkaline phosphatase

Test	Latest Reference Range and Units	Patient’s Lab
WBC	3.8-11.2 x 10^9^/L	5.3
Hemoglobin	13.4-17.2 g/dL	13.8
Hematocrit	39.5-49.7%	41.2
Platelet count	150-450 x 10^9^/L	237
PT	11.6-14.2 sec	13.1
PTT	25-36 sec	32
Sodium	133-145 mmol/L	136
Potassium	3.3-5.1 mmol/L	5.1
Chloride	96-108 mmol/L	103
CO_2_	21-31 mmol/L	20
Anion gap	9-18 mmol/L	13
BUN	8-24 mg/dL	30
Creatinine	0.80-1.40 mg/dL	1.51
Glucose	70-99 mg/dL	128
Calcium	8.6-10.3 mg/dL	9.3
Total protein	5.9-8.4 g/dL	7.9
Albumin	3.5-5.2 g/dL	4.7
A/G ratio	1.3-2.7	1.5
Globulin	2.0-3.6 g/dL	3.2
AST (SGOT)	14-43 U/L	20
ALT (SGPT)	12-58 U/L	9
Alk phos	40-118 U/L	71
Bilirubin, total	0-1.2 mg/dL	0.4

An initial ECG demonstrated a WCT, as shown in Figure 1, which was successfully terminated using pharmacological cardioversion with IV amiodarone, restoring sinus rhythm, as illustrated in Figure 2.

**Figure 1:**
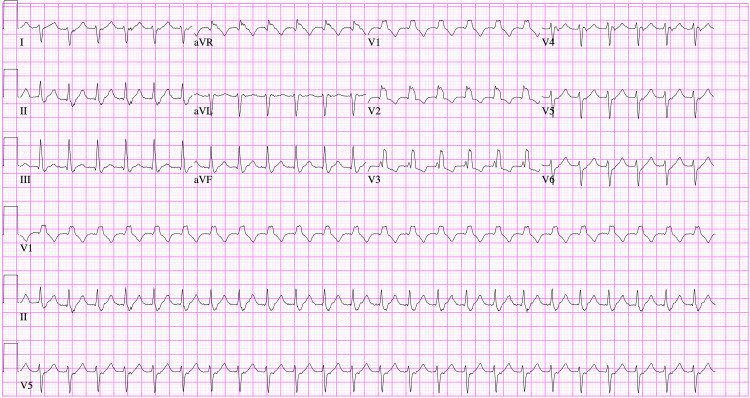
Wide complex tachycardia demonstrating right bundle branch block morphology and rightward axis deviation, with a QRS axis of 122°.

**Figure 2:**
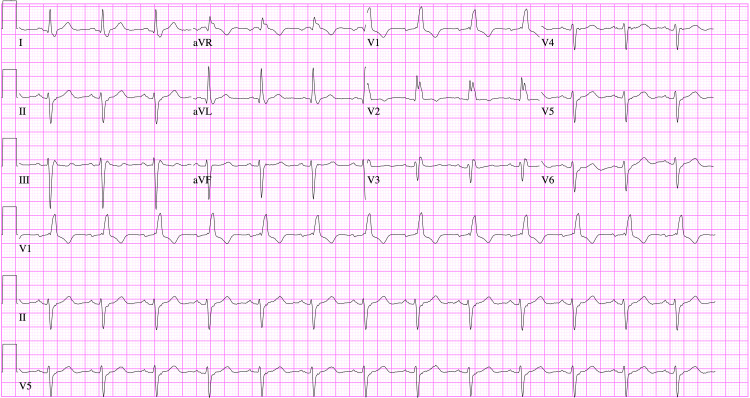
Baseline sinus rhythm electrocardiogram demonstrating right bundle branch block and leftward axis deviation, with a QRS axis of -62°.

Is this WCT consistent with VT? How can these findings be explained?

## Discussion

As described in the clinical presentation, the utility of physical and systemic examination is significant with limits for arrhythmia approach. To differentiate between VT and SVT, there have been no formally established guidelines or findings to aid the diagnosis. The presence of the “frog sign,” rapid neck pounding owing to simultaneous activation of both atrium and ventricle, has been described to support the diagnosis of AVNRT [8-10]. However, it is illogical to rely on this sign given the absence of a dedicated study on its sensitivity, specificity, positive predictive value, and negative predictive value. Further, the “absence” of the “frog sign,” as in our case, does not completely rule out the diagnosis of AVNRT. Therefore, ECG is inevitably paramount to help discern the diagnosis. Overall, the ECG demonstrated a WCT, for which the principal differential diagnoses include VT and SVT with aberrant conduction. Given the patient’s advanced age and history of prior myocardial infarction, VT was initially considered the most likely diagnosis.

The tachycardia exhibited a right bundle branch block (RBBB) morphology with a relatively preserved QRS duration compared with the baseline RBBB (Figure 1). However, a new rightward axis deviation was observed, which differed significantly from the leftward axis during sinus rhythm (Figure 2). In this context, left anterior fascicular VT was considered a potential diagnosis, and the tachycardia was subsequently converted to sinus rhythm with pharmacologic cardioversion using intravenous amiodarone in the ED.

Subsequently, the patient was admitted for further evaluation and initiated on extended-release verapamil 240 mg daily with continuous telemetry monitoring. No recurrence of WCT was observed during hospitalization, and the patient was discharged with outpatient follow-up and a planned EP study with consideration for VT ablation.

In the interim, the patient developed another episode of WCT with leftward axis deviation, which was successfully terminated with electrical cardioversion (Figure 3).

**Figure 3:**
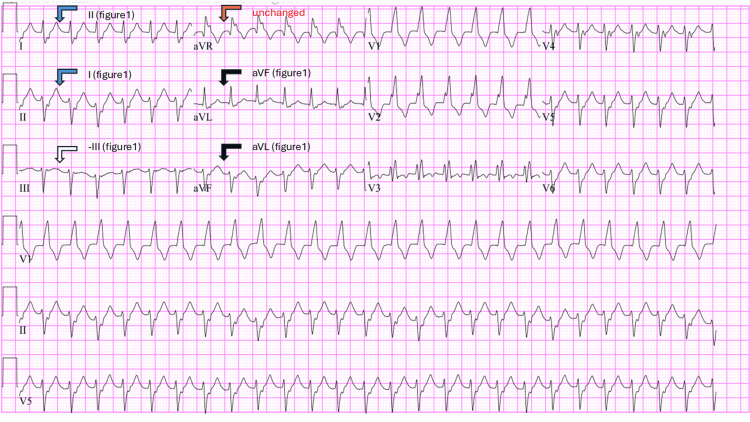
Recurrent wide complex tachycardia demonstrating leftward axis deviation with a QRS axis of -85° (black arrows) and precordial morphology identical to that observed during sinus rhythm. Compared with Figure 1, the black arrows demonstrate reversal of polarity between leads aVL and aVF; the blue arrows demonstrate reversal between leads I and II; the white arrow demonstrates inverted polarity in lead III; and the red arrow demonstrates preserved vector orientation in lead aVR.

Although fascicular VT remained a possibility, it would be highly unusual - though not impossible - for a single patient to exhibit tachycardias consistent with both left anterior fascicular VT (Figure 1) and left posterior fascicular VT (Figure 3). Furthermore, there were no definitive ECG features such as atrioventricular dissociation, capture beats, or fusion beats to strongly support a VT diagnosis. Non-fascicular VT, particularly originating from the interventricular septum or conduction system, also remained a consideration [11]. Closer inspection revealed a discrepancy in the limb leads between Figures 1, 3, while the precordial leads remained identical. Leads I, II, III, aVL, and aVF were inverted (Figure 3; arrows) relative to one another, and further, a resultant lead axis changed, portrayed in Figures 1, 3, from rightward to leftward axis, respectively, whereas lead aVR remained unchanged. This pattern raised suspicion for limb lead reversal and is consistent with left arm-left leg (LA to LL) lead reversal [11].

With LA to LL reversal, the most pronounced and easiest to recognize changes are to lead III, lead aVL, and aVF. The polarities in these leads would be opposite to the corrected placement, while the configuration between lead I and lead II is swapped. Accordingly, the lead axis, which typically can be defined using lead I and aVF, would have shifted. As a result, the new axis would be derived from the original lead II and aVL, as described above, explaining the observation of axis reformation between Figures 1, 3.

When applying established VT-SVT discrimination tools, including the Brugada [6] and Basel [7] algorithms, the diagnosis could not be reliably reconciled. Firstly, as demonstrated in Figures 1, 3, precordial leads V3-V6 exhibited RS complexes, none of which exceeded 100 ms. Secondly, no apparent atrioventricular dissociation was observed on either tracing. Thirdly, the QRS morphology in leads V1 and V6 appeared atypical for RBBB. Collectively, these findings increased the likelihood of VT; however, interpretation of the final step of the Brugada criteria may remain subject to interobserver variability.

Similarly, the probability of VT remained elevated, as the clinical presentation fulfilled at least two of the three Basel criteria, including a prior history of myocardial infarction and a time to first peak exceeding 40 ms in lead aVR. Nevertheless, measurement of the time to first peak in leads aVR and II may be susceptible to overestimation or underestimation in the presence of baseline artifact. Furthermore, the absence of fusion or capture beats on the available tracings did not provide sufficient confidence to exclude VT, although neither did it constitute definitive evidence supporting SVT.

Upon further review, comparison of the WCT ECGs (Figures 1, 3) with the baseline sinus rhythm ECG (Figure 2) demonstrated that the baseline ECG closely resembled the WCT morphology seen in Figure 1.

Given these findings, the patient underwent a comprehensive EP study to definitively establish the diagnosis and guide management. The study demonstrated intact anterograde and retrograde conduction via the atrioventricular node, with a clear atrio-His (AH) interval jump observed during atrial double extrastimulus testing (600/400 ms). Parahisian pacing confirmed the absence of an accessory pathway (Figure 4).

**Figure 4:**
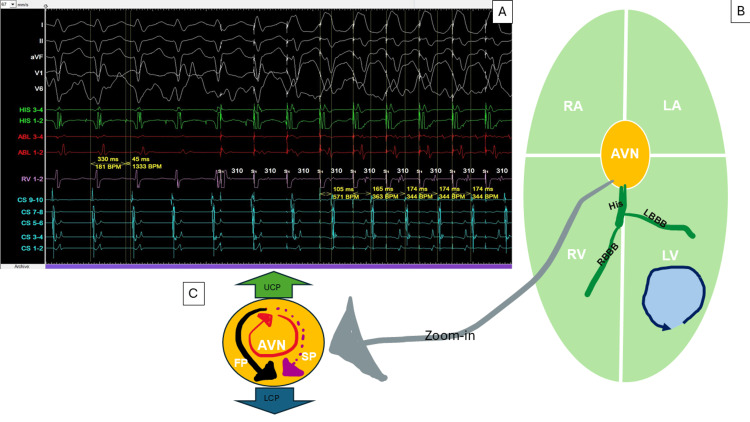
Intracardiac electrograms. (A) Intracardiac recordings showing inducible AVNRT with VA = 0 and termination by ventricular overdrive pacing. (B) Schematic illustration of the normal cardiac conduction system, demonstrating propagation from AVN to His bundle, giving rise to RBBB and LBBB, along with the proposed reentrant circuit for VT (blue arrow). (C) Enlarged schematic representation of typical AVNRT (red arrow), formed by the SP and FP. Conduction subsequently propagates to the atrium via the UCP and to the ventricle via the LCP. AVN: atrioventricular node; AVNRT: atrioventricular nodal reentrant tachycardia; FP: fast pathway; LA: left atrium; LBBB: left bundle branch block; LCP: lower common pathway; LV: left ventricle; RA: right atrium; RBBB: right bundle branch block; RV: right ventricle; SP: slow pathway; UCP: upper common pathway; VT: ventricular tachycardia. Original image created by the authors using Microsoft PowerPoint (Microsoft Corp., Redmond, WA, USA).

The WCT was reproducibly inducible (Figure 4A), with simultaneous atrial and ventricular activation (VA = 0) and an identical ECG pattern to that shown in Figure 1. The tachycardia cycle length was 375 ms, with an AH interval greater than the His-atrial (HA) interval. Ventricular overdrive pacing consistently terminated the tachycardia and demonstrated a late transition zone. These findings were diagnostic of typical AVNRT with RBBB aberrancy [12].

Radiofrequency ablation targeting the right inferior extension of the slow pathway was subsequently performed, consisting of nine applications with a total ablation time of 8.2 minutes. During ablation, slow junctional beats with preserved antegrade and retrograde conduction were observed. At the conclusion of the procedure, repeat atrial extrastimulus testing no longer demonstrated an AH jump. The tachycardia was no longer inducible, with reproducible noninducibility demonstrated both in the presence and absence of isoproterenol infusion, including during the washout period. No recurrence of SVT has been observed during five months of follow-up. The timeline of the presentation and management is shown in Figure 5.

**Figure 5:**
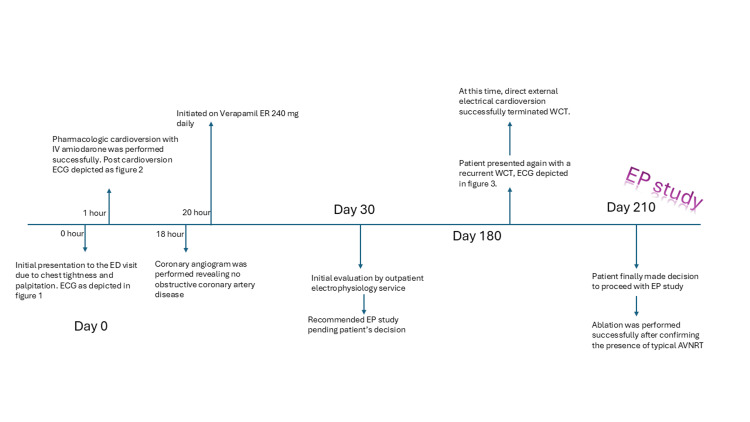
Timeline of the patient’s presentation and management. IV: intravenous; ED: emergency department; EP: electrophysiology; WCT: wide complex tachycardia; AVNRT: atrioventricular nodal reentrant tachycardia; ER: extended-release Original image created by the authors using Microsoft PowerPoint (Microsoft Corp., Redmond, WA, USA).

Limitations

The present report has several limitations. First, the inherent limitations and potential biases associated with a single-patient case report are unavoidable. Second, recognition of the limb lead reversal was made retrospectively rather than during the patient’s initial presentation. Third, the currently available follow-up data remain limited to the short term; therefore, longer-term follow-up is warranted to more definitively exclude the possibility of VT.

## Conclusions

This case highlights the diagnostic challenge in distinguishing WCT due to SVT with aberrancy from VT. Ancillary information, including baseline ECG morphology and careful assessment of lead placement, can provide critical diagnostic clues. Awareness of limb lead reversal is essential, as it may significantly alter ECG interpretation and mimic malignant arrhythmias. Nevertheless, while surface ECG analysis may increase diagnostic confidence, invasive EP study remains the gold standard for definitive diagnosis and appropriate treatment of complex WCTs.

Some of the key learning points were to always compare WCT morphology with the patient’s baseline sinus-rhythm ECG. Discrepancies between limb leads while precordial leads remain identical should raise suspicion for limb-lead reversal. LA-LL reversal inverts III and swaps I/II (aVR unchanged). Axis analysis becomes unreliable until lead placement is verified. Empirical VT-specific therapy in stable WCT should be chosen cautiously; verapamil in non-fascicular VT can be hemodynamically catastrophic. Surface-ECG algorithms have real limits; invasive EP study remains the definitive arbiter in complex WCT.
